# Economic costs of obesity in Thailand: a retrospective cost-of-illness study

**DOI:** 10.1186/1472-6963-14-146

**Published:** 2014-04-02

**Authors:** Paiboon Pitayatienanan, Rukmanee Butchon, Jomkwan Yothasamut, Wichai Aekplakorn, Yot Teerawattananon, Naeti Suksomboon, Montarat Thavorncharoensap

**Affiliations:** 1 Social and Administrative Pharmacy Division, Department of Pharmacy, Faculty of Pharmacy, Mahidol University, Bangkok, Thailand; 2 Health Intervention and Technology Assessment Program (HITAP), Ministry of Public Health, Nonthaburi, Thailand; 3 Faculty of Medicine, Ramathibodi Hospital, Mahidol University, Bangkok, Thailand; 4 Clinical Pharmacy Division, Department of Pharmacy, Faculty of Pharmacy, Mahidol University, Bangkok, Thailand

**Keywords:** Cost-of-illness, Obesity, Overweight, Thailand, Economic

## Abstract

**Background:**

Over the last decade, the prevalence of obesity (BMI ≥ 25 kg/m^2^) in Thailand has been rising rapidly and consistently. Estimating the cost of obesity to society is an essential step in setting priorities for research and resource use and helping improve public awareness of the negative economic impacts of obesity. This prevalence-based, cost-of-illness study aims to estimate the economic costs of obesity in Thailand.

**Methods:**

The estimated costs in this study included health care cost, cost of productivity loss due to premature mortality, and cost of productivity loss due to hospital-related absenteeism. The Obesity-Attributable Fraction (OAF) was used to estimate the extent to which the co-morbidities were attributable to obesity. The health care cost of obesity was further estimated by multiplying the number of patients in each disease category attributable to obesity by the unit cost of treatment. The cost of productivity loss was calculated using the human capital approach.

**Results:**

The health care cost attributable to obesity was estimated at 5,584 million baht or 1.5% of national health expenditure. The cost of productivity loss attributable to obesity was estimated at 6,558 million baht - accounting for 54% of the total cost of obesity. The cost of hospital-related absenteeism was estimated at 694 million baht, while the cost of premature mortality was estimated at 5,864 million baht. The total cost of obesity was then estimated at 12,142 million baht (725.3 million US$PPP, 16.74 baht =1 US$PPP accounting for 0.13% of Thailand’s Gross Domestic Product (GDP).

**Conclusions:**

Obesity imposes a substantial economic burden on Thai society especially in term of health care costs. Large-scale comprehensive interventions focused on improving public awareness of the cost of and problems associated with obesity and promoting a healthy lifestyle should be regarded as a public health priority.

## Background

Obesity (defined as having a Body Mass Index (BMI) greater than or equal to 30 Kilogram (Kg)/Meter(M)^2^) [1], is a growing health concern worldwide. It is a known risk factor for a number of chronic diseases including cardiovascular diseases, diabetes, musculoskeletal disorders, and some cancers [2-5]. Aside from increased morbidity, obesity has also been found to increase premature mortality [6-8], decrease productivity due to absenteeism and presenteeism [9], and decrease quality of life [10-13].

As a result of increasing global urbanisation, changes in dietary habits, and declining levels of physical activity, the obesity epidemic is no longer limited to populations in Europe and North America [14-17]. Today, it affects populations in most countries, including those in Latin America and Asia. According to the World Health Organisation (WHO), the global prevalence of obesity has more than doubled between 1980 and 2008 [18]. In 2008, the WHO estimated that more than 1.4 billion adults aged 20 and over were overweight (a BMI greater than or equal to 25 Kg/M^2^) [18]. Of these overweight adults, 500 million were obese [18].

The economic cost of obesity is substantial. According to one estimate from a recent systematic review, the health costs associated with obesity may account for between 0.7% and 2.8% of a country’s total health care expenditure [19]. In another review of data from ten Western European countries, estimated obesity costs were found to be as high as 0.09% to 0.61% of Gross Domestic Product (GDP) [20]. Estimating the cost of obesity to society is critical for policy makers, public health planners, and other health stakeholders. Not only can the cost estimate be used to establish priorities for research and health resource use, but it can also be used to improve public awareness of the negative economic impacts of obesity. In the past, many attempts have been made to estimate the economic cost of obesity in western countries [21-24]; few studies of this kind were conducted in Asia. However, in light of the rapid and continuous increase in obesity prevalence, several countries in Asia including Korea [25], Taiwan [26], China [27], and Hong Kong [28] have begun to assess the economic cost of obesity.

In line with global trends, the prevalence of obesity in Thailand almost doubled between 1991 and 2009. According to the fourth National Health Examination Survey (NHES) 2008-9 [29], 28.4% of adult Thai men and 40.1% of women were classified as obese (BMI ≥ 25 Kg/M^2^). More importantly, the NHES also found that obesity levels had risen disproportionately in rural areas [30], indicating that obesity was no longer only found in higher socioeconomic groups. Despite this rapid increase in obesity over the last ten years, no research has yet been conducted into the economic cost of obesity in Thailand. Our study aims to estimate the economic costs of obesity in Thailand, 2009.

## Methods

This is a prevalence-based, cost-of-illness study. Costs included in the analyses were health care cost, cost of productivity loss due to premature mortality, and cost of productivity loss due to hospital-related absenteeism.

### Obesity and co-morbidities

In this study, obesity is defined as having a BMI of 25 Kg/m^2^ or higher. To estimate the cost associated with obesity, a number of co-morbidities were identified and their respective costs calculated. Based on the degree of association with obesity, the availability of existing information and its importance in the Thai context, the following 12 co-morbidities were selected in our study: colon and colorectal cancer, breast cancer, endometrial cancer, hyperlipidemia, diabetes mellitus, depression, hypertension, ischemic heart disease, pulmonary embolism, stroke, gall bladder disease, and osteoarthritis. For each co-morbidity, the Obesity Attributable Fraction (OAF), the proportion of the incidence of a co-morbidity in the population that is due to obesity, was calculated using the following formula [31]:

$$\mathrm{OA}F_{j}=\frac{\sum_{i=1}^{2}P_{i}\left(RR_{\mathrm{ij}}-1\right)}{\sum_{i=0}^{2}P_{i}\left(RR_{\mathrm{ij}}-1\right)+1}$$

Where

i = Body Mass Index (BMI) level (i = 1 means BMI ≥ 25.0-29.9 kg/m^2^ and i = 2 means BMI ≥ 30 kg/m^2^)

j = Co-morbidity related to obesity (j = 1 -12)

P_i_ = Prevalence of obesity at BMI level i

RR_ij_ = Relative Risk of co-morbidity j associated with obesity level i compared with the non-obese population

In this study, obesity prevalence (P_i_) was obtained from the 4th NHES [17] while the Relative Risks (RR_ij_) were derived from meta-analyses [32-34] as well as studies conducted in Asia [26,35].

### Health care cost

The health care costs of obesity and the 12 co-morbidities were estimated for both inpatient and outpatient services. For each co-morbidity, the inpatient and outpatient healthcare costs attributable to obesity were calculated by multiplying the total number of patients with the given co-morbidity in Thailand by the corresponding OAF, and the average cost of each co-morbidity per person per year. Each co-morbidity total was then added together to give a total healthcare cost for obesity.

The outpatient data on the total number of patients and the data on the cost of outpatient visit(s) for each co-morbidity per person per year in 2009 were obtained from the database of the Center for Health Equity Monitoring (CHEM), Faculty of Medicine, Naresuan University. This database includes outpatient information covered by the two major public health insurance schemes in Thailand—the Universal Coverage Scheme (UCS) and the Civil Medical Service Scheme (CSMBS)—from 675 out of 843 public hospitals (80%) across all 76 provinces throughout the country. These two public health schemes cover approximately 80% of the total Thai population (approximately 67 million); the remaining 20% are covered by the Social Security Scheme (SSS), which is offered to formal private sector employees. To estimate the total number of outpatient visits in Thailand in 2009, it was assumed that 64% of total outpatient visits in Thailand would be those covered by the CHEM database.

The inpatient data on the total number of patients and the data on the cost of inpatient visit(s) for each co-morbidity per person per year were obtained from the Central Office for Health care Information (COHI) database, 2009, which contains hospital admission data from all public hospitals for patients covered by the UCS and CSMBS (but not the SSS). It was assumed that the COHI data would represent 80% of total inpatients in Thailand.

### Cost of productivity loss due to premature mortality

The costs associated with productivity loss due to premature mortality were calculated for each co-morbidity using the human capital approach. The number of deaths that could be attributed to obesity in 2009, disaggregated by age and gender, were multiplied by the average wage each person would receive if he or she lived through his or her lifespan. A discount rate of 3% was employed [29]. The data on the total number of deaths from each co-morbidity were obtained from the 2004 Thai Burden of Disease (BOD) project, and data on average earnings were calculated from the 2009 National Economic and Social Survey.

### Cost of productivity loss due to hospital related absenteeism

The cost of hospital-related absenteeism was also calculated using the human capital approach. To estimate the cost of productivity loss due to hospital related absenteeism, the number of days that inpatients and outpatients with obesity-related conditions were absent from work in 2009 as a result of their obesity was multiplied by the average daily wage. Outpatient absentee data was obtained from the CHEM database; inpatient absentee data was obtained from the COHI database. The calculation was based on the assumption that the average outpatient visit took 0.5 days. The average daily wage was calculated by dividing Thailand’s 2009 GDP per capita [36] by the number of working days in the same year.

## Results

The overall relative risk estimates and OAFs for obesity and the 12 co-morbidities, disaggregated by gender, are presented in Table 1. OAF estimates indicate that about 24% to 52% of all cases of diabetes mellitus, 25% to 33% of all cases of ischemic heart disease, and 15% to 23% of all cases of osteoarthritis in Thailand are attributable to obesity, respectively.

**Table 1:** Relative risks for selected co-morbidities in obese subjects and Obesity Attributable Fraction (OAF)

**Diseases/conditions**	**Relative risk of developing diseases**				**Obesity attributable fraction (OAF) (%)**	
	**Male**		**Female**		**Male**	**Female**
	1*	2**	1*	2**		
Breast cancer [32]	-	-	1.08	1.13	-	2
Colon and colorectal cancer [32]	1.51	1.95	1.45	1.66	8	9
Depression [32]	1.30	1.31	0.98	1.67	4	3
Diabetes mellitus [32]	2.40	6.47	3.92	12.41	24	52
Endometrial cancer [32]	-	-	1.53	3.22	-	17
Gall bladder [32]	1.09	1.43	1.44	2.32	2	12
Hyperlipidemia [26]	1.95	1.76	1.95	1.76	11	15
Hypertension [32]	1.28	1.84	1.65	2.42	5	15
Ischemic heart disease [35]	3.02	4.37	3.02	4.37	25	33
Obesity	1.00	1.00	1.00	1.00	100	100
Osteoarthritis [32]	2.76	4.20	1.80	1.96	23	15
Pulmonary embolism [32]	1.91	3.51	1.91	3.51	15	22
Stroke [32]	1.23	1.51	1.15	1.49	4	5

*1 = BMI25.0-29.9 kg/m^2^ **2 = BMI ≥ 30 kg/m^2^*.*

Estimates of the overall economic costs of obesity, disaggregated by types of cost, gender, and co-morbidity are displayed in Table 2. With regard to total cost, the three conditions that are found to incur the highest costs are diabetes mellitus (6,385.7 million baht), ischemic heart disease (2,168.4 million baht), and stroke (2,017.6 million baht).

**Table 2:** Estimates of the economic costs of obesity in Thailand 2009 by types of costs, gender, and co-morbidity

**Disease**	**Health care cost (Million baht)**		**Cost of premature mortality (Million baht)**		**Cost of productivity loss due to hospital-related absenteeism (Million baht)**		**Total cost (Million baht)**		
	**Male**	**Female**	**Male**	**Female**	**Male**	**Female**	**Male**	**Female**	**All**
Diabetes mellitus	663.8	2,722.8	1,302.6	1,247.6	88.1	360.7	2,054.5	4,331.2	6,385.7
Ischemic heart disease	521.5	549.1	761.6	273.3	29.6	33.2	1,312.7	855.7	2,168.4
Stroke	98.9	99.6	1,236.1	564.5	9.2	9.4	1,344.1	673.5	2,017.6
Colon and rectal cancer	188.0	189.3	203.5	119.0	6.0	6.3	397.4	314.7	712.1
Hypertension	31.4	146.4	26.5	26.1	18.6	83.7	76.5	256.2	332.7
Osteoarthritis	46.3	113.6	0.7	0.6	8.2	17.6	55.2	131.8	187
Gall bladder	11.5	101.0	**-**	**-**	1.1	8.9	12.6	109.9	122.5
Endometrial cancer	-	42.3	-	2.8	-	2.5	0	47.6	47.6
Breast cancer	-	36.6	-	99.3	-	1.7	0	137.6	137.6
Obesity	3.8	4.6	**-**	**-**	1.2	2.5	5.0	7.1	12.1
Hyperlipidemia	0.9	2.1	**-**	**-**	1.1	2.5	2.0	4.6	6.6
Pulmonary embolism	1.8	5.9	**-**	**-**	0.1	0.5	1.9	6.5	8.4
Depression	0.8	1.8	**-**	**-**	0.4	0.7	1.3	2.5	3.8
**Total**	1,568.7	4,015.1	3,531.0	2,333.2	163.6	530.2	5,263.2	6,878.9	12,142.1

As shown in Table 2, the estimated health care cost attributable to obesity is 5,584 million baht. Obesity-related health care costs for women are about 2.5 times higher than for men (4,015 million baht VS 1,569 million baht). The three conditions that incur the highest health care costs are diabetes mellitus (3,386.6 million baht), ischemic heart disease (1,070.6 million baht), and colorectal cancer (377.3 million baht).

The estimated cost of premature mortality as a result of obesity-related conditions is 5,864 million baht. The premature mortality costs incurred by men are 1.5 times higher than they are in women (3,531 million baht VS 2,333 million baht). The three conditions that incur the highest premature mortality costs are diabetes mellitus (2,550.2 million baht), stroke (1,800.6 million baht), and ischemic heart disease (1,034.9 million baht).

The estimated cost of productivity loss due to absenteeism as a result of obesity-related conditions is 694 million baht. Of this total, 448.8 million baht results from diabetes mellitus, 102.3 million baht from hypertension, and 62.8 million baht from ischemic heart disease.

A summary of all of the estimated costs attributable to obesity is presented in Table 3. The total estimated economic cost of obesity in Thailand is 12,142.1 million baht (725.3 million US$PPP, 16.74 baht =1 US$PPP [36] or 0.13% of GDP [36]. Health care costs account for 46% of the total cost or about 1.5% of the national health care expenditure [37], while productivity loss costs account for 56% of the total cost.

**Table 3:** Summary of the estimated economic costs of obesity in Thailand 2009

**Cost**	**Million baht**	**%**
Direct cost (health care cost)	5,584	46
OPD	850	
IPD	4,734	
Indirect cost (productivity loss)	6,558	54
Premature mortality	5,864	
Hospital-related absenteeism	694	
Total cost	12,142	100
% of total cost in term of GDP	0.13	
% of Health care cost in term of National health care expenditure [37]	1.5	

## Discussion

Many studies have shown that obesity exerts a significant cost burden on a country’s health system and productivity [19,20]. This was also found in this first analysis of obesity cost in the Thai context, where obesity-attributable costs were found to be substantial, accounting for 0.13% of GDP or 1.5% of the total national health expenditure. In addition, the analysis revealed that costs associated with heath care provision and costs associated with productivity loss were broadly similar, which are in line with the findings of previous studies [22,38,39]. The cost identified in this paper should be regarded as a minimum estimate since other related costs such as the cost of absenteeism not related to hospitalization, cost of presenteeism, and unemployment costs were not included in the analysis. Furthermore, due to the unavailability of data in Thailand, the cost of premature mortality due to obesity and the following five co-morbidities—gall bladder disease, obesity, hyperlipidemia, pulmonary embolism, and depression—were not included in the analysis.

A WHO report [18] found that, globally, obesity and overweight account for 23% of coronary heart disease cases, 7-14% of cancer cases, and 44% of diabetes mellitus cases. These general proportions were also found in our analysis, which suggested that about 25-33% of ischemic heart cases, 2% of breast cancer cases, 17% of endometrial cancer cases, 8-9% of colon cancer cases, and 24-52% of diabetes mellitus cases in Thailand were associated with obesity. Unlike in Western countries [21-23], our study did not find cardiovascular disease related to obesity to be the primary leading cause of economic burden. In line with a previous study in Asia [25], and giving weight to recent concerns that have been voiced regarding the epidemic of obesity and type 2 diabetes in Asia [16], we found diabetes mellitus to be the first leading cause of obesity cost (6,385.7 million baht), followed by ischemic heart disease (2,168.4 million baht), and stroke (2017.6 million baht). Nevertheless, previous studies indicate [40,41] that reducing weight by 5–10% can improve blood sugar control and help reduce the risk of developing cardiovascular disease. Given the rise of obesity in Asia, and the prevalence of related conditions—particularly diabetes mellitus and cardiovascular disease [16]—interventions aimed at obesity control clearly deserve more attention.

In line with findings from previous studies in the US [6,42,43], which found that obesity had a health impact equal or exceeding that of smoking and drinking, our results indicate that health care costs attributable to obesity are the same as those attributable to alcohol consumption, which was estimated at 5,491 million baht in 2006 [44]. Despite this, the numbers of public health campaigns targeting obesity are fewer than those related to smoking and drinking. One explanation may be that obesity and the condition of being overweight are perceived as personal issues rather than social problems. However, our findings clearly show that the effect of obesity on the country’s economy is significant, especially in terms of health care costs, which are currently shouldered by all tax payers in Thailand. It is clear that, to effectively tackle obesity in Thailand, a public health campaign targeting obesity epidemic should place emphasis on the impact of obesity on society as well as social responsibility without stigmatising those who are obese.

In this study, BMI was used as a measure to determine the prevalence of obesity. According to the WHO [45], a BMI reading of 25-29.9 kg/m^2^ is indicative of an overweight condition, while a reading of 30 kg/m^2^ or higher indicates obesity. However, in Asia, the risk of type 2 diabetes mellitus and cardiovascular disease is already high in those whose BMI is below 25 kg/m^2^. In addition, at the same BMI, Asian populations are found to have higher levels of body fat than Western populations [46,47]. Therefore, it has been proposed that lower BMI readings should be used to identify those who are overweight or obese in the Asian population. A 2004 WHO expert consultation proposed that an appropriate cut off to measure the condition of being overweight and obese in Asian populations would be 23-24.9 kg/m^2^ and 25 kg/m^2^, respectively [47]. To permit comparison across previous studies in estimating the economic cost of obesity, a BMI reading of at least 25 kg/m^2^ was used to define obesity in our study. Nevertheless, it should be noted that the estimate impact of obesity will be lower if a BMI reading of 30 kg/m^2^ is used to define obesity.

In this study, the prevalence of obesity in 2009 was used to calculate the OAF. The estimated prevalence constitutes people with a varied time period of obesity. As induction times for chronic diseases may differ across persons and diseases and are not exactly known, we might have overestimated the cost from the impact of obesity as the lapse time need for developing comorbidity as well as duration of obesity were not taken into account. Nevertheless, these figures do inform at what cost the societal inevitably need to shoulder in the future without effective interventions to mitigate the current burden of obesity. In addition, the identification of induction times for chronic diseases is a priority area for future research related to obesity.

Another limitation that warrants further discussion is the reliance on estimated costs. While we acknowledge that the validity of our findings relies on the accuracy of a number of estimated parameters and assumptions, we are confident that the estimates are reasonably accurate. For instance, the CHEM and COHI databases (which were used to estimate health care costs) are the largest hospital databases available in Thailand. Nevertheless, we assumed that the COHI accounted for 80% of all inpatients in the country. However, based on the recent figures [48], patients in COHI database may account for 83% of the total population. Therefore, our results might slightly overestimate the impact of obesity. In addition, while these databases only include data from patients who are covered by the UCS and CSMBS schemes, we assume that obesity prevalence among beneficiaries of these two schemes will be comparable to those of the SSS. We acknowledge, however that this assumption may be somewhat limited as the SSS scheme covers those who are healthy enough to be employed in the private sector; this may mean that they suffer from lower levels of obesity. If this is the case, then the total cost of obesity would be somewhat lower than what we have estimated in this study. Lastly, since there was no Thai-specific relative risk data available, the relative risk data were obtained from meta-analysis review of global literature, including a number of Asian studies [26,32-35]. Future research on relative risk in Thailand, particularly for diabetes and cardiovascular disease, would be beneficial.

## Conclusions

This study confirmed that obesity imposes a substantial economic burden on Thai society. In terms of health care cost, it is equivalent to that imposed by alcohol consumption. In light of the rapid and continuous increase in obesity prevalence in Thailand, large-scale comprehensive interventions for the prevention and control of obesity should be regarded as of public health priority in Thailand.

## Competing interests

The authors declare that they have no competing interests.

## Authors’ contributions

PP participated in study design, data collection, data analysis, and drafted the manuscript. NS participated in its design and coordination, data collection, and data analysis. RB participated in its design and data analysis. JY participated in its design and data collection. WA participated in its design, data collection and data analysis. YT conceived of the study, participated in its design and data analysis, MT conceived of the study, participated in its design and coordination, data collection, data analysis, and helped to draft the manuscript. All authors read and approved the final manuscript.

## Pre-publication history

The pre-publication history for this paper can be accessed here:

http://www.biomedcentral.com/1472-6963/14/146/prepub

## References

[B1] Obesity and overweight [http://www.who.int/mediacentre/factsheets/fs311/en/] Accessed: March 2013

[B2] Burton BT Foster WR Hirsch J Vanlttallie TB Health implication of obesity: NIH consensus development conference Int J Obes Relat Metab Disord 1985 14 155 169 3840463

[B3] Janssen I Katzmarzyk PT Ross R Body mass index, waist circumference, and health risk: evidence in support of current National Institutes of Health guidelines Arch Intern Med 2002 14 2074 2079 12374515 10.1001/archinte.162.18.2074

[B4] Must A Spadano J Coakley EH FiIeld AE CoLditz G Dietz WH The disease burden associated with overweight and obesity JAMA 1999 14 1523 1529 10546691 10.1001/jama.282.16.1523

[B5] Pi-Sunyer FX Medical hazards of obesity Ann Intern Med 1993 14 655 660 8363192 10.7326/0003-4819-119-7_part_2-199310011-00006

[B6] Jia H Lubetkin EI Trends in quality-adjusted life-years lost contributed by smoking and obesity Am J Prev Med 2010 14 138 144 20117569 10.1016/j.amepre.2009.09.043

[B7] Jia H Lubetkin EI Obesity-related quality-adjusted life years lost in the U.S. from 1993 to 2008 Am J Prev Med 2010 14 220 227 20709253 10.1016/j.amepre.2010.03.026

[B8] Mokdad AH Marks JH Stroup DF Gerberding JL Actual causes of death in the United States, 2000 J Am Med Assoc 2004 14 1238 1245 10.1001/jama.291.10.1238 15010446

[B9] Gates DM Succop P Brehm BJ Gillespie GL Sommers BD Obesity and presenteeism: the impact of body mass index on workplace productivity J Occup Environ Med 2008 14 39 45 18188080 10.1097/JOM.0b013e31815d8db2

[B10] Jia H Lubetkin EI The impact of obesity on health-related quality-of life in the general adult US population J Public Health 2005 14 156 164 10.1093/pubmed/fdi025 15820993

[B11] Larsson U Karlsson J Sullivan M Impact of overwieght and obesity on health-related quality of life- a Swedish population study Int J Obes Relat Metab Disord 2002 14 417 424 11896499 10.1038/sj.ijo.0801919

[B12] Sach TH Barton GR Doherty M Muir KR Jenkinson C Avery AJ The relationship between body mass index and health-related quality of life: comparing the EQ-5D, EuroQol VAS and SF-6D Int J Obes (Lond) 2007 14 189 196 16682976 10.1038/sj.ijo.0803365

[B13] Wee HL Wu Y Thumboo J Lee J Tai ES Assocation of body mass index with Short-Form 36 physical and mental component summary scores in a multiethinic Asian population Int J Obes (Lond) 2010 14 1034 1043 20142824 10.1038/ijo.2010.24

[B14] Asia Pacific Cohort Studies Collaboration The burden of overweight and obesity in the Asia-Pacific region Obes Rev 2007 14 191 196 10.1111/j.1467-789X.2006.00292.x 17444961

[B15] Ramachandran A Snehalatha C Rising burden of obesity in Asia J Obesity 2010 14 1 8 10.1155/2010/868573 PMC2939400 20871654

[B16] Yoon KH Lee JH Kim JW Cho JH Choi YH Ko SH Zimmet P Son HY Epidemic obestiy and type 2 diabetes in Asia Lancet 2006 14 1681 1688 17098087 10.1016/S0140-6736(06)69703-1

[B17] Misra A Khurana L Obesity and the metabolic syndrome in developing countries J Clin Endocrinol Metab 2008 14 Suppl 1 9 30 10.1210/jc.2008-1595 18987276

[B18] World Health Organization Obesity and Overweight 2011 Geneva: World Health Organization

[B19] Withrow D Alter DA The economic burden of obesity worldwide: a systematic review of the direct costs of obesity Obesity Rev 2011 14 131 141 10.1111/j.1467-789X.2009.00712.x 20122135

[B20] Müller-Riemenschneider F Reinhold T Berghöfer A Willich SN Health-Economic burden of obesity in Europe Eur J Epidemiol 2008 14 499 509 18509729 10.1007/s10654-008-9239-1

[B21] Birmingham CL Muller JL Palepu A Spinelli JJ Anis AH The cost of obesity in Canada CMAJ 1999 14 483 488 10081464 PMC1230073

[B22] Sander B Bergemann R Economic burden of obesity and its complications in Germany Eur J Health Econ 2003 14 248 253 15609192 10.1007/s10198-003-0178-1

[B23] Swinburn B Ashton T Gillespie J Cox B Menon A Simmons D Birkbeck J Health care costs of obesity in New Zealand Int J Obes Relat Metab Disord 1997 14 891 896 9347407 10.1038/sj.ijo.0800486

[B24] Wolf AN Colditz G Social and economic effects of body weight in the United States Am J Clin Nutr 1996 14 Suppl 3 466 469 10.1093/ajcn/63.3.466 8615344

[B25] Kang JH Jeong BG Cho YG Song HR Kim KA Socioeconomic costs of overweight and obesity in Korean adults J Korean Med Sci 2011 14 1533 1540 22147988 10.3346/jkms.2011.26.12.1533 PMC3230011

[B26] Fu T Wen T Yeh P Chang C Costs of metabolic syndrome-related diseases induced by obesity in Taiwan Obesity Rev 2008 14 Suppl 1 68 73 10.1111/j.1467-789X.2007.00441.x 18307702

[B27] Zhao W Zhai Y Hu J Wang J Yang Z Kong L Chen C Economic burden of obesity-related chronic diseases in Mainland China Obes Rev 2008 14 Suppl 1 62 67 18307701 10.1111/j.1467-789X.2007.00440.x

[B28] Ko GTC The cost of obesity in Hong Kong Obes Rev 2008 14 Suppl 1 74 77 18307703 10.1111/j.1467-789X.2007.00442.x

[B29] Permsuwan U Guntawongwan K Buddhawongsa P Handling time in economic evaluation studies J Med Assoc Thai 2008 14 Suppl 2 53 58 19255986

[B30] Aekplakorn W Mo-Suwan L Prevalence of obesity in Thailand Obes Rev 2009 14 589 592 19656310 10.1111/j.1467-789X.2009.00626.x

[B31] Walter SD Estimation and interpretation of attributable risk in health research Biometrics 1976 14 829 849 1009228

[B32] Guh DP Zhang W Bansback N Amarsi Z Birmingham CL Anis AH The incidence of co-morbidities related to obesity and overweight: a systematic review and meta-analysis BMC Public Health 2009 14 88 1 20 10.1186/1471-2458-9-88 PMC2667420 19320986

[B33] Luppino FS de Wit LM Bouvy PF Stijnen T Cuijpers P Penninx BW Zitman FG Overweight, obesity, and depression: a systematic review and meta-analysis of longitudinal studies Arch Gen Psychiatry 2010 14 220 229 20194822 10.1001/archgenpsychiatry.2010.2

[B34] Whitlock G Lewington S Sherliker P Clarke R Emberson J Halsey J Qizilbash N Collins R Peto R Prospective Studies Collaboration Body-mass index and cause-specific mortality in 900 000 adults: collaborative analyses of 57 prospective studies Lancet 2009 14 1083 1096 19299006 10.1016/S0140-6736(09)60318-4 PMC2662372

[B35] Jee SH Sull JW Park J Lee SY Ohrr H Guallar E Samet JM Body-mass index and mortality in Korean men and women N Engl J Med 2006 14 779 787 16926276 10.1056/NEJMoa054017

[B36] World Economic Outlook Database: September 2011 [http://www.who.int/mediacentre/factsheets/fs311/en/] Accessed: March 2013

[B37] The 2009-2010 NHA working group National Health Accounts of Thailand 2009-2010 2012 Nonthaburi, Thailand: International Health Policy Program, Ministry of Public Health

[B38] Ahn B Hyojee J Socioeconomic cost of obesity in Korea Korean Nutrition Soc 2005 14 786 792

[B39] Wolf AN Colditz GA Current estimates of the economic costs of obesity in the United States Obes Res 1998 14 97 106 9545015 10.1002/j.1550-8528.1998.tb00322.x

[B40] Bosello O Armellini F Zamboni M Fitchet M The benefits of modest weight loss in type II diabetes Int J Obes Relat Metab Disord 1997 14 Suppl 1 10 13 9130035

[B41] Van Gaal LF Wauters MA De Leeuw IH The beneficial effects of modest weight loss on cardiovascular risk factors Int J Obes Relat Metab Disord 1997 14 Suppl 1 5 9 9130034

[B42] Sturm R The effects of obesity, smoking, and drinking on medical problems and costs Health Affairs 2002 14 245 253 11900166 10.1377/hlthaff.21.2.245

[B43] Sturm R Wells KB Does obesity contribute as much to mortality as poverty or smoking? Publ Hlth 2001 14 229 235 10.1038/sj/ph/1900764 11429721

[B44] Thavorncharoensap M Teerawattananon Y Yothasamut J Lertpitakpong C Thitiboonsuwan K Neramitpitagkul P Chaikledkaew U The economic costs of alcohol consumption in Thailand, 2006 BMC Public Health 2010 14 323 20534112 10.1186/1471-2458-10-323 PMC2896941

[B45] WHO Expert Committee Physical Status: the Use and Interpretation of Anthropometry 1995 Geneva 8594834

[B46] Durenberg P Yap M van Staveren WA Body mass index and percent body fat: a meta-analysis among different ethnic groups Int J Obesity 1998 14 1164 1171 10.1038/sj.ijo.0800741 9877251

[B47] WHO Expert consultation Appropriate body-mass index for Asian populations and its implications for policy and intervention strategies Lancet 2004 14 157 163 10.1016/S0140-6736(03)15268-3 14726171

[B48] Thammatach-aree J Health Systems, Public Health Programs, and Social Determinants of Health: Thailand 2011 Rio de Janeiro, Brazil: Proceeding of the World Health Organization for the World Conference on Social Determinants of Health

